# The Convallis Rule for Unsupervised Learning in Cortical Networks

**DOI:** 10.1371/journal.pcbi.1003272

**Published:** 2013-10-24

**Authors:** Pierre Yger, Kenneth D. Harris

**Affiliations:** UCL Institute of Neurology and UCL Department of Neuroscience, Physiology, and Pharmacology, London, United Kingdom; University of Oxford, United Kingdom

## Abstract

The phenomenology and cellular mechanisms of cortical synaptic plasticity are becoming known in increasing detail, but the computational principles by which cortical plasticity enables the development of sensory representations are unclear. Here we describe a framework for cortical synaptic plasticity termed the “Convallis rule”, mathematically derived from a principle of unsupervised learning via constrained optimization. Implementation of the rule caused a recurrent cortex-like network of simulated spiking neurons to develop rate representations of real-world speech stimuli, enabling classification by a downstream linear decoder. Applied to spike patterns used in *in vitro* plasticity experiments, the rule reproduced multiple results including and beyond STDP. However STDP alone produced poorer learning performance. The mathematical form of the rule is consistent with a dual coincidence detector mechanism that has been suggested by experiments in several synaptic classes of juvenile neocortex. Based on this confluence of normative, phenomenological, and mechanistic evidence, we suggest that the rule may approximate a fundamental computational principle of the neocortex.

## Author Summary

The circuits of the sensory cortex are able to extract useful information from sensory inputs because of their exquisitely organized synaptic connections. These connections are wired largely through experience-dependent synaptic plasticity. Although many details of both the phenomena and cellular mechanisms of cortical synaptic plasticity are now known, an understanding of the computational principles by which synaptic plasticity wires cortical networks lags far behind this experimental data. In this study, we provide a theoretical framework for cortical plasticity termed the “Convallis rule”. The computational power of this rule is demonstrated by its ability to cause simulated cortical networks to learn representations of real-world speech data. Application of the rule to paradigms used to probe synaptic plasticity in vitro reproduced a large number of experimental findings, and the mathematical form of the rule is consistent with a dual coincidence detector mechanism that has been suggested experimentally in juvenile neocortex. Based on this confluence of normative, phenomenological, and mechanistic evidence, we suggest that the rule may approximate a fundamental computational principle of the neocortex.

## Introduction

Animal learning is believed to occur primarily through changes in synaptic strengths. Experimental work has revealed an increasingly detailed picture of synaptic plasticity [1], [2], at the level of both phenomenology and cellular mechanisms. However an understanding of synaptic plasticity's computational role in cortical circuits lags far behind this experimental knowledge. While spike timing dependent plasticity (STDP) has gained much attention, the STDP rule is simply a description of how synapses respond to one particular paradigm of temporally offset spike pairings, and is neither a complete description of synaptic behaviour, nor a computational principle that explains how learning could occur in cortex [3]–[6]. It therefore seems likely that STDP is just an approximation to a more fundamental computational principle that explains the form and function of cortical synaptic plasticity. Such a principle would not only have to be consistent with experimental results on the phenomena and mechanisms of synaptic plasticity, but also explain why it provides a computational benefit. A strong test of the latter is whether simulated cortex-like circuits employing the same principle can learn to perform real-world information processing tasks.

The nature and mechanisms of synaptic plasticity differ between brain regions, developmental stages, and cell types, likely indicating different computational roles of synaptic plasticity in different contexts. In the sensory cortex, synaptic plasticity is strongest at early ages [7], and is believed to play an important role in the development of sensory representations. The juvenile cortex learns to form representations of sensory stimuli even in the absence of any required behavior or reward: the acquisition of native language sounds, for example, begins through passive exposure to speech before infants can themselves speak [8]. The outcome of such learning is not simply a more faithful representation of the learned stimuli — which are already faithfully represented by sensory receptors themselves — but a transformation of this representation into a form where relevant information can be more easily read out by downstream structures [9]. This problem of forming easily-decoded representations of a data set, without reward or training signals, is called “unsupervised learning” [10], [11].

Unsupervised learning has long been proposed as a primary function of the sensory cortex [12], [13]. An intriguing connection between cortical plasticity and artificial algorithms for unsupervised learning arises from work of Bienenstock, Cooper, and Munro (BCM) [14]. A key feature of the BCM rule is that inputs occurring when the postsynaptic firing rate is below a “plasticity threshold” will be weakened, whereas inputs firing when postsynaptic firing rate exceeds the plasticity threshold will be strengthened; the rule is made stable by allowing the plasticity threshold to “slide” as a function of mean postsynaptic activity. The BCM rule operates at the level of firing rate neurons, and at this level has been successful in modelling a number of experimental results such as the development of visual receptive fields [15]. Theoretical analysis [16] has shown that this scheme allows simplified neuron models to implement an unsupervised learning algorithm similar to projection pursuit [17] or independent component analysis (ICA) [18], [19], extracting non-Gaussian features of their inputs which are *a priori* more likely than Gaussian features to correspond to signals of interest.

Although the BCM theory was originally defined at the level of firing rates, more recent modeling work [20]–[24] has reproduced a dependence of the direction of synaptic plasticity on postsynaptic firing rate in spike-based neurons. In cortical neurons synaptic plasticity depends not only of postsynaptic firing rates, but also shows a similar dependence on subthreshold depolarization, with presynaptic spikes during strong postsynaptic depolarizations leading to potentiation, and during weak postsynaptic depolarization leading to depression [25], [26]. Computational models incorporating such behavior have successfully matched several experimental findings of *in vitro* plasticity [23].

In the present work, we present a framework for unsupervised learning in cortical networks. The rule is derived as an optimization of the skewness of a cell's postsynaptic membrane potential distribution under a constraint of constant firing rate, and leads to a voltage-dependence similar to that observed experimentally [25]. We term the resulting framework the Convallis rule after the Latin word for “valley”, in reference to the shape of the voltage objective function. We show that the Convallis rule causes simulated recurrent spiking networks to perform unsupervised learning of speech sounds, forming representations that enable a downstream linear classifier to accurately identify spoken words from the spike counts of the simulated neurons. When presented with paired pre- and postsynaptic spikes or other paradigms used *in vitro*, predictions of the Convallis rule more accurately match experimental results than the predictions of STDP alone. Furthermore, simulation of STDP alone (or of previously published plasticity rules [21], [23]) produced poorer performance on speech learning than the full Convallis rule, indicating that STDP may be just one signature of a cortical plasticity principle similar to Convallis. The mathematical form of the Convallis rule suggests implementation by a dual coincidence detector mechanism, consistent with experimental data from juvenile sensory cortex [6], [27]–[33].

## Results

We derived the Convallis rule from two principles, analogous to those underlying artificial unsupervised learning algorithms such as ICA. The first principle is that synaptic changes should tend to increase the skewness of a neuron's subthreshold membrane potential distribution. Because the physical processes that produce structure in real-world data sets often show substantial higher-order moments, whereas random and uninformative combinations follow a Gaussian distribution, projections with non-Gaussian distribution are *a priori* more likely to extract useful information from many real-world data sets [19]. The second principle is that despite synaptic plasticity, neurons should maintain a constant average firing rate. This principle is required for stable operation of the rule, and is again analogous to a step of the ICA algorithm (see below).

**Figure 1:**
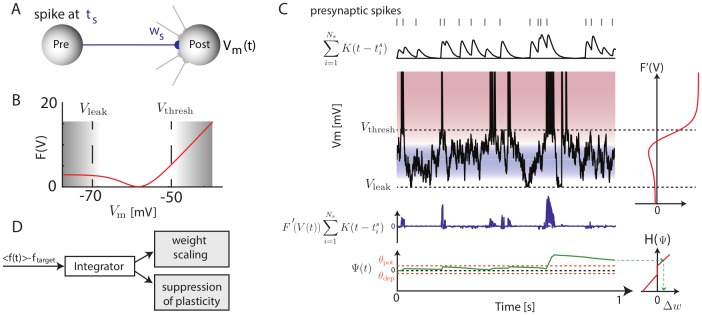
Illustration of the Convallis rule. (**A**) Schematic of a particular plastic synapse (blue) onto a post-synaptic neuron with membrane potential 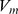. (**B**) The objective function 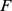 optimized by the neuron: 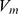 values in between resting state or threshold are penalized, while values close to rest or spike threshold are rewarded. (**C**) Illustration of the learning rule. Presynaptic spike times (top, gray lines), are filtered by the EPSP shape 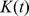 (top black trace). This activity is multiplied by 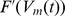 (shown to the right of 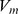), to yield a function that is positive when the presynaptic cell fires shortly before 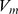 is close to threshold, and negative for presynaptic spikes at intermediate 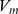 (blue trace). This function is then accumulated through a slowly decaying exponential (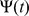, green, bottom), and passed through a shrinkage function 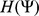 (right) to yield the weight changes. The horizontal orange lines indicate the thresholds 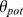 and 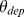 that 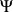 must cross to yield potentiation and depression. (**D**) Illustration of the rate constraint mechanism. Deviations of the long-run average firing rate from a target value lead to multiplicative scaling of excitatory synaptic inputs and suppression of activity-dependent plasticity until the rate target is restored.

To derive the rule, we first defined an objective function 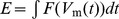 that measures the non-Gaussianity of the subthreshold distribution. The function 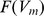 has the valley-shaped form shown in Figure 1B. Optimization of this objective function ensures that the postsynaptic neuron spends as much time as possible close to either resting potential or spiking threshold, but as little time as possible in a zone of intermediate membrane potential, i.e. exhibiting a skewed, non-Gaussian subthreshold distribution. The form of 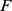 used in simulations is described in the Materials & Methods, although our results did not depend critically on this precise formula (data not shown).

To implement the first principle of skewness optimization, we first compute the derivative of this objective function with respect to the neuron's input weights. Making certain assumptions (see Materials and Methods for a full derivation) we obtain:
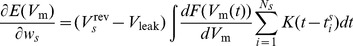
(1)where 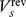 is the reversal potential of synapse 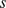, 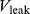 is the rest voltage of the neuron, 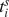 are the times of action potentials incoming onto synapse 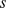, and 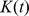 is the shape of a postsynaptic potential elicited by synapse 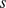. When a presynaptic input fires shortly before the neuron is close to spiking threshold, the integrand is positive leading to an increase in synaptic weight, but when a presynaptic neuron fires shortly prior to a potential only just above rest the integrand is negative leading to a decrease in synaptic weight. This voltage dependence is similar to that observed experimentally in cortical neurons [25] and also employed in previous phenomenological models [23]. We note that a direct computation of this integral would be computationally prohibitive, as it would require numerical solution of a differential equation for every synapse and at every time step of the simulation. Tractable simulation of this rule was however made possible by a trick that enabled solution of only a single differential equation per neuron (see Materials and Methods). In our simulations, voltage was reset to a level of −55 mV after action potential firing, followed by an afterdepolarization simulating the effects of active dendritic conductances [34] (see Materials and Methods). This reset mechanism, rather than the reset to rest commonly employed in integrate-and-fire simulations, was necessary in order to produce voltage traces similar to those seen in experimental recordings of cortical pyramidal cells (see Figure S1), and also played an important role in matching *in vitro* plasticity results (see below).

While equation 1 is sufficient to implement our first principle of skewness optimization, we found that better learning performance, as well as a closer match to physiological data, could be obtained with an additional feature modeled after the statistical technique of shrinkage [11]. Specifically, the integrand of equation 1 was not used directly to modify weights, but first convolved with a decaying exponential to yield a function 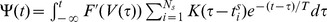, and then passed through a nonlinear shrinkage function 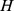 to ensure plasticity only occurs in response to multiple coincidences: 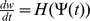 ([22], [24], [35]; see Materials and Methods for more details). This ensures that weight changes occur only due to reliable and repeated relationships between presynaptic activity and postsynaptic membrane potentials, rather than random occurrence of single spikes. An illustration of how pre- and post-synaptic activity lead to weight changes under this rule is shown in Figure 1C. Physiologically, such an integration mechanism could be instantiated via self-exciting kinases as suggested previously [22].

The second principle underlying the Convallis rule is a constraint on the mean firing rate of each neuron to a target value. Analogous principles are also often found in machine learning algorithms: in ICA, for example, the root-mean-square activity of each unit is fixed at a constant value by a constraint on the weight vector norm together with sphering of inputs [19]. Such constraints are typically implemented in one of two ways: by including a penalty term in the objective function, whose gradient is then added to the learning rule resulting in “weight decay”; or by repeated projection of the system parameters onto a subspace satisfying the constraint [19]. In our simulations, we found that simple gradient ascent was not effective at enforcing stability, and therefore used a projection method. This was implemented by a mechanism which responded to deviations from the target firing rate by linearly scaling all excitatory synaptic weights up or down [36], and suppressing activity-dependent plasticity until the rate constraint was restored (Figure 1D; see Materials and Methods for details). Physiologically, the “metaplasticity” [37], [38] required for suppression of synaptic changes until rate homeostasis is restored, could be instantiated via one of the many molecular pathways gating induction and expression of synaptic plasticity.

**Figure 2:**
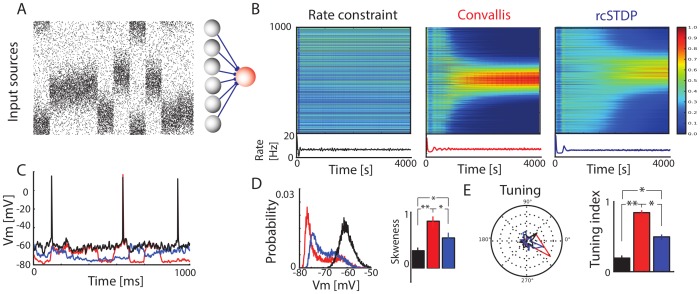
Operation of the Convallis rule in a simple feed-forward situation. (**A**) The activity of a population of input neurons was simulated by ascribing each input a location on a virtual circle. Every 100 ms, the firing rate of the inputs was updated as a circular Gaussian distribution with random center, and spike trains were simulated as Poisson processes with this rate. These inputs were fed to a single output cell that employed rate constraint alone, the Convallis rule, or STDP together with rate constraint (rcSTDP). (**B**) Evolution of the input weights and mean firing rate of an example neuron during learning. Note the development of spatially selective inputs for the Convallis rule, but not rate constraint, and the development of approximate selectivity by rcSTDP. (**C**) Illustrative membrane potential trace after learning in the three different conditions. (**D**) Probability distribution of the membrane potential for the neurons shown in **B**, and skewness values averaged over a population of 1000 neurons. (**E**) Tuning for the neurons shown in **B**, and tuning index values averaged over a population of 1000 neurons. Black bars and traces represent rate constraint rule only; red represents Convallis rule; and blue represents rcSTDP. Error bars show standard error of the mean. * represents 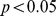, t-test, and ** represents 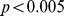.

To study the rule's effects, we first considered the behaviour of an individual neuron implementing the rule on a simple artificial data set. The parameters used in the learning rule were fixed in this and all subsequent simulations (see Materials and Methods for more details). For this first artificial task, inputs consisted of a population of 1000 excitatory sources (see Figure 2A). The simulated postsynaptic neuron received plastic excitatory synapses from these sources, as well as constant inhibitory background with input at 10 Hz through 250 synapses which were not subject to plasticity. We first considered a simple case where inputs fired as Poisson spike trains with rates determined as spatial Gaussian profiles whose centre changed location every 100 ms (Figure 1A; see Materials and Methods) [21], [22], [39]. When weights evolved according to the rate constraint only, no structure was seen in the weight patterns. With the Convallis rule, postsynaptic neurons developed strong weights from groups of closely-spaced and thus correlated inputs, but zero weights from neurons uncorrelated with this primary group. When weights instead evolved by classical all-to-all STDP augmented by the rate constraint (called rcSTDP, see Materials and Methods for details), the firing rate was kept at the desired value of 10 Hz, and weights became more selective, but in a manner less closely related to the input statistics. Examination of post-synaptic voltage traces showed that after learning with the Convallis rule, but not after rate constraint alone, the membrane potential spent considerably longer close to resting potential (Figure 2C), corresponding to an increased skewness of the membrane potential histogram, (Figure 2D; 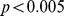, t-test). This in turn reflected the development of selectivity of the neurons to particular stimuli (Figure 2E) (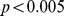, t-test). Application of rcSTDP caused an increase in skewness tuning intermediate between rate constraint alone and the Convallis rule, even after optimizing by parameter search (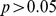, t-test; see Figure S2). This confirms that the Convallis rule is able to perform unsupervised learning in a simple artificial task, causing neurons to select inputs from groups of coactive neurons; STDP produces a poorer approximation to the same behavior.

**Figure 3:**
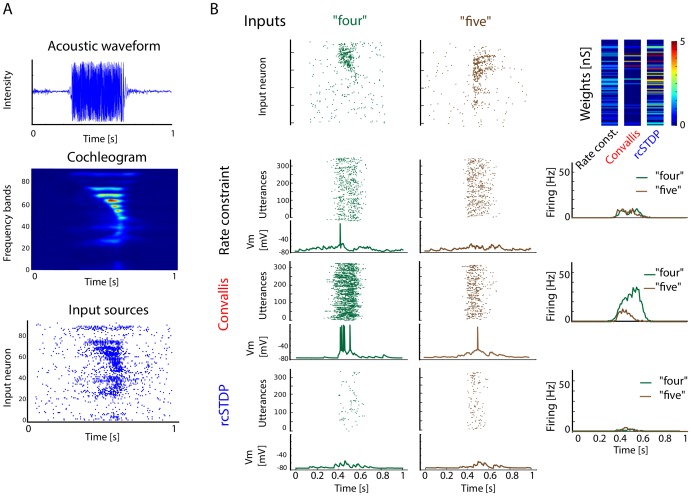
Illustration of Convallis rule as applied to speech data. (**A**) Preprocessing pipeline. Waveforms corresponding to utterances of eleven spoken digits (zero to nine plus “oh”) by multiple speakers were processed by a cochleogram model [41], which was used to produce inhomogeneous Poisson spike trains of 100 input cells. (**B**) Illustration of spiking and voltage responses after learning for two particular digits. Top row: examples of the input population spike patterns corresponding to a single presentation of the digits “four” and “five”. Top row right, pseudocolor representation of the simulated neuron's input weights after learning, for rate constraint, the Convallis rule, and rcSTDP. Bottom three rows show a raster representation of the trained neuron's responses to a test set consisting of 300 utterances of these digits by previously unheard speakers, together with a membrane potential trace from a single test-set utterance. Right column shows mean firing rate vs. time averaged over the whole test set, illustrating the development of selective responses by the Convallis rule.

We next asked whether the Convallis rule would enable individual simulated neurons to perform unsupervised learning in a real-world problem. Because we are interested in the development of cortical representations of sensory stimuli, we asked whether the Convallis rule could promote unsupervised formation of representations of speech sounds. Spike train inputs were generated from the TIDIGITS database of spoken digits [40], by pre-processing with a cochlear model filter bank [41], followed by transformation into inhomogeneous Poisson spike trains that contacted the simulated neuron with a range of synaptic delays (Figure 3A; see Materials and Methods). Figure 3B (top row) shows a representation of the output of the cochleogram for utterances of the digits “four”, and “five”. To the right is a pseudocolor representation of the excitatory weights developed by neurons initialized to random weights and trained on 326 utterances of all digits by the rate constraint mechanism alone, by the Convallis rule, or by rcSTDP. Each digit was repeated ten times. Figure 3B (lower three rows) shows the response of these three neurons to a test set consisting of previously unheard utterances of the same digits by different speakers. The neuron trained by Convallis responds selectively to “four” while the response to “five” is largely eliminated, whereas the neuron trained by rate constraint alone responds equally to both. Thus, the Convallis rule has enabled the neuron to develop a differential response to the presented digits, which has generalized to utterances of the same digits spoken by new speakers.

**Figure 4:**
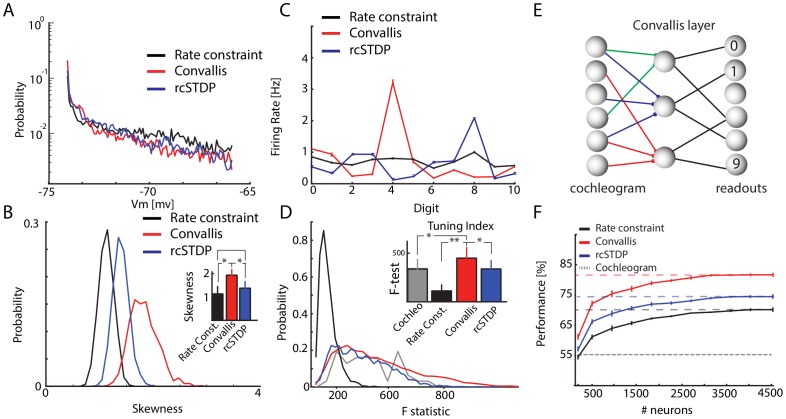
Feed-forward processing of speech data. (**A**) Histogram of subthreshold potentials for the cell illustrated in Figure 3, accumulated over all test-set data after learning with three different plasticity rules. (**B**) Distribution of skewness for 4500 neurons trained similarly from random initial weights. Skewness after Convallis training is significantly higher than after rate constraint or rcSTDP, but rcSTDP and rate constraint do not differ. (**C**) Mean rate response of the example neuron to all digits. Errors bar show s.e.m. (**D**) The strength of tuning for each neuron was summarized by an F-statistic that measured the selectivity of its spike counts for particular digits (see Materials and Methods). The main graph shows an histogram of tuning strength across the simulated population for the 3 learning rules and the raw cochleogram input, while the inset shows mean and standard error. Note that while the Convallis rule produces sharper tuning than the cochleogram inputs, rate constraint alone and rcSTDP produce weaker tuning. (**E**) To evaluate the ability of these rules to perform unsupervised learning, the spike count responses of up to 4500 cells were used as input to a linear classifier trained to distinguish digits. (**F**) Mean classification performance as a function of the number of unsupervised neurons. (Errors bars show s.e.m over 10 independent runs of the analysis). The left axis marks the number of neurons in the cochleogram representation, and the horizontal dashed line indicates classification from the raw cochleogram. Note that Convallis outperforms the raw cochleogram, populations trained by rate constraint, and populations trained by rcSTDP for all numbers of neurons.

To verify that this behaviour holds in general, we performed five thousand independent simulations of the Convallis rule in single neurons, with excitatory and inhibitory inputs drawn from the simulated cochlear cells, each trained by 10 presentations of the TIDIGITS training set, which we found sufficient to ensure convergence of all learning rules (Figure S3). Each simulation began from a different random weight configuration. The mean firing rate constraint was fixed to 1.5 Hz for all cells. As previously seen with artificial inputs, the membrane distribution produced in response to this real-world input was more skewed after training with the Convallis rule (Figure 4A for the example cell shown in Figure 3, Figure 4B for population summary). On average, over 1000 independent runs, there was a significant difference in skewness between Convallis and rate constraint alone, with rcSTDP producing an intermediate increase in skewness (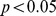). We measured the selectivity of the simulated neurons using an F-statistic that measured differences in spike count between different digits (see Materials and Methods). The Convallis rule caused neurons to become more selective (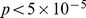, t-test), whereas application of rate constraint alone or rcSTDP led to output neurons that were actually less selective than the raw cochleogram input (Figures 4C for the same example cell shown in Figure 3, Figure 4D for population average). Similar results were found when comparing Convallis to multiple implementations of the STDP rule as well as for other plasticity rules described in the modelling literature [21], [23] (see Figure S4).

The aim of unsupervised learning is to generate representations of input data that enable downstream neurons to easily form associations with them. Although complete information about the stimulus is of course present in the raw input, a downstream cell may not be able to extract this information unless it is represented in a suitable form. We next asked whether the representation generated by the Convallis rule allowed improved classification by a linear downstream readout in which spike timing information was discarded; this choice was motivated by results indicating that information in higher sensory cortices can be progressively more easily read out in such a format [9]. Specifically, we used a linear support vector machine to predict which digit was uttered, from the spike counts of a population of simulated cells arranged in a feedforward configuration (Figure 4E; see Materials and Methods; note that while the SVM was trained with a biologically unrealistic quadratic programming algorithm, the same solution would be found by a large-margin perceptron [42]). Figure 4F shows the generalization performance of the classifier (measured on the TIDIGITS test set) as a function of population size. Performing the classification from a layer of neurons that used rate constraint alone produced an improvement over prediction directly from the cochleogram. The size of this improvement increased with the number of cells used, consistent with reports that large numbers of random projections can provide useful data representations [43], [44]. Applying the Convallis rule produced a substantially improved representation over the rate constraint alone (18% vs 29.9% errors; 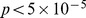, t-test), whereas rcSTDP produced an intermediate improvement (25.9% error; 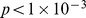, t-test). Evaluation of performance with time-reversed digit stimuli indicated that the neurons had learned specific temporal features of the input rather than simply frequency content (Figure S3). Evaluation of several other proposed learning rules for spiking neurons taken from the literature, such as rcNN-STDP (STDP with interactions only between neighbouring pairs of spikes, and the rate constraint), triplet STDP [21] with rate constraint, or phenomenological rules also based on post-synaptic voltages [23] (see Materials and Methods for details) also confirmed that their performance did not match those of the Convallis rule (25.0%, 27% and 25.9% vs 18.0% errors; see Figure S4).

**Figure 5:**
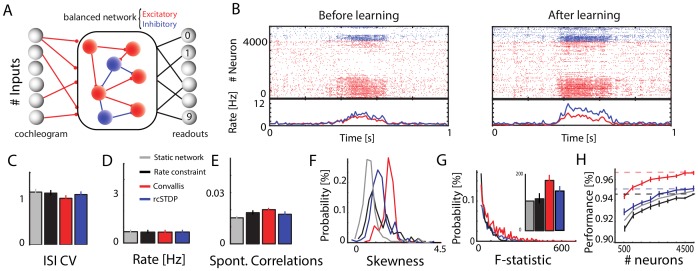
Learning and classification in a balanced recurrent network. (**A**) Network illustration. A set of 3600 excitatory and 900 inhibitory recurrently connected neurons are driven by an external excitatory input drawn from a cochleogram simulated as before. Excitatory synapses within the network are plastic while inhibitory synapses and external inputs are kept fixed. A population of linear readout neurons use the spike counts of the recurrent excitatory neurons to classify the spoken digits. (**B**) Illustration of population activity in the network, before and after learning, in response to a particular digit. The rasters show activity of the entire population of excitatory and inhibitory neurons (red and blue) to a single digit presentation; the lower curves show population-averaged firing rate throughout this trial. Note that training produces no visible change in global network dynamics, which maintains an asynchronous regular state. (**C–E**) Distributions of ISI CVs, firing rates, and pairwise correlation coefficients (averaged over 2000 randomly chosen pairs of cells) in the network before and after learning with rate constraint only, rcSTDP, or with the Convallis learning rule. Note that none of the learning rules produce a change in any of these measures of network dynamics. Error bars show the standard deviation. (**F**) Distribution of membrane potential skewness for 200 randomly chosen cells in the network before or after learning. Note that skewness is highest with the Convallis rule. (**G**) Distribution of the tuning sharpness (as measured by F-statistic) for all neurons before and after learning. Inset displays the mean of the distributions. Error bars show standard deviation. (**H**) Classification performance as a function of the number of neurons considered by the external classifier. Errors bars show s.e.m over 10 different simulations, run independently from different random seeds. While Convallis learning produces improved performance, rate constraint did not, and rcSTDP produced a smaller but still significant improvement (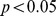).

The above analysis showed that the Convallis rule caused individual neurons to develop selective representations of the digit stimuli, which when arranged together in a feedforward configuration formed a population code that enabled the spoken digit to be decoded with 82% accuracy. The cortex, however, is a recurrent rather than a feedforward network, and we next asked whether a recurrent architecture would lead to further improved classification performance (Figure 5A). Recurrent spiking network models can exhibit multiple global patterns of population activity, of which the asynchronous irregular state provides the closest match to *in vivo* cortical activity in alert animals [45]–[47]. We set the initial conductances (prior to training) to obtain asynchronous irregular activity at a mean spontaneous activity at 1.5 Hz, and with the coefficient of variation of inter-spike intervals (CV ISI) equal to 1.1 (Figure 5C; see Materials and Methods). When a sound input was presented to the network, mean firing rates increased from 1.5 Hz to 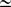 15 Hz (Figure 5B), while remaining in the asynchronous irregular regime.

To measure the ability of the Convallis rule to produce unsupervised learning in recurrent spiking networks, we trained the network with 10 iterations of the TIDIGITS training set, which were again sufficient for convergence (see Figure S5). All recurrent excitatory connections in the network were plastic, while inhibitory and input connections were fixed. Running the learning rule did not disrupt the asynchronous irregular dynamics of the network, as indicated by the ISI CV, mean firing rate distribution, and mean spontaneous correlation values (Figure 5B and Figure 5C, D, E). As in the feed-forward case, the network's constituent neurons showed increased tuning and membrane potential skewness after training (Figure 5F, G).

The ability to perform unsupervised learning in a recurrent network was again measured by ability to identify the spoken digits using a linear classifier trained on the spike counts of the network's excitatory neurons (Figure 5H). We note that even prior to training, as in the feed-forward case, the representation generated by the recurrent network allowed higher classification performance than the raw cochleogram input (5.8% error), consistent with previous reports that randomly connected “liquid-state” networks can compute useful representations of spatiotemporal input patterns [48]–[50]. Training with the Convallis rule significantly boosted performance to reach 3.3% error (Figure 5H). As in the feedforward case, application of rcSTDP produced error rates more than 50% higher than those of the full Convallis rule (Figure 5H) (5.1% error; 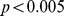). Thus, the Convallis rule enables spiking neurons to perform unsupervised learning on real-world problems, arranged either in a feedforward or in a recurrent configuration. As in the feed-forward scenario, performance with time-reversed digit stimuli indicated that the neurons had learned specific temporal features of the input rather than simply frequency content (Figure S5). Once again, we were unable to produce comparable results with rules previously published in the literature, which resulted in error rates more than 50% higher than those produced by Convallis (5.2% and 5.3% errors for rcNN-STDP and rcTriplet, respectively; see Figure S6).

**Figure 6:**
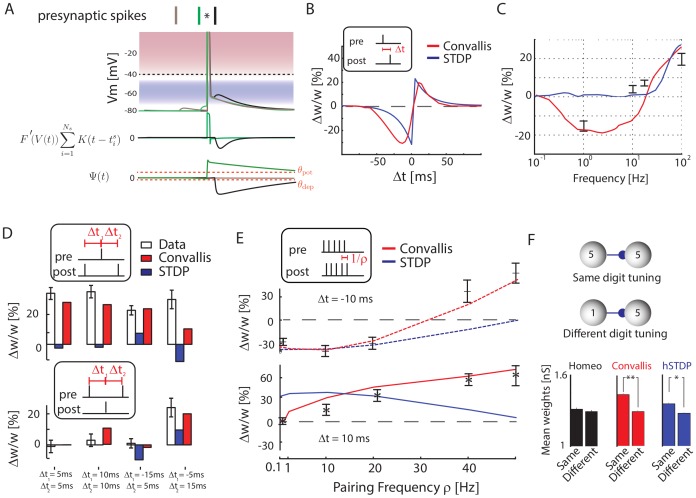
Reproduction of experimental findings. (**A**) Schematic illustrating the Convallis learning rule in case of a 10 ms post-pre (black), a 10 ms pre-post pairing (green), or a 30 ms pre-post pairing (brown). (**B**) Synaptic modifications arising after 60 spike pairings repeated at 1 Hz, as a function of time 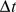 between pre- and post-synaptic spikes. The red curve indicates the results of the Convallis rule, the blue curve indicates the traditional bi-exponential STDP curve for comparison purposes. (**C**) Effect of tetanic stimuli at various frequencies. Red curve indicates Convallis rule results, errorbars are data reproduced from [55]. For the Convallis rule, as for the original data, high frequency stimulation yields potentiation, intermediate frequency stimulation yields depression, whereas the lowest frequencies yield no effect. (**D**) Effect of post-pre-post (top) and pre-post-pre (bottom) spike triplets at various intervals. White bars represent data from [52], red represents Convallis simulation, blue represents STDP. (**E**) Effect of repeating post-pre (top) and pre-post (bottom) pairings at frequencies between 0.1 and 50 Hz. Errorbars indicate data from [26], red indicates Convallis simulation and blue indicates STDP. (**F**) After training synapses are stronger between neurons representing similar features, as found experimentally in mouse cortex [57]. Histograms show mean synaptic weights after training the recurrent network on speech sounds, (Figure 5) for neuronal pairs maximally responsive to the same (top schematic, example of two neurons both tuned to digit 5), or different digits (bottom schematic, two neurons tuned to different digits). * represents 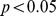, t-test, and ** represents 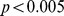.

The Convallis rule was derived mathematically from an optimization principle, rather than by fitting to experimentally measured parameters. Before suggesting that an analogous process might occur in the cortex, it is thus important to check how a neuron employing this rule would behave in paradigms that have been used to experimentally probe cortical synaptic plasticity. Although we found simulation of rcSTDP alone produced poorer learning than Convallis, STDP is a robustly observed experimental result that the Convallis rule must reproduce if a similar rule does occur in cortical neurons. To test this, we applied a spike-pairing paradigm to two simulated cells, using the same parameters as in the previous speech-classification simulations. Figure 6A shows a close-up view of the Convallis rule in operation for three spike pairings. The green trace shows a pre-post interval of 10 ms. Here, the period immediately after the presynaptic spike (where 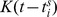 is positive) contains an action potential, leading to a high value of 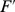, and synaptic potentiation. The black trace shows a post-pre pairing of −10 ms. In this case, the period immediately following the presynaptic spike occurs during the postsynaptic afterdepolarization, a moderately depolarized voltage range for which 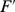 is negative. The gray trace shows a pre-post interval of 30 ms, longer than the duration of the kernel 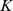. Now, the postsynaptic potential during the entire period while 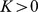 is very close to rest, leading to a value of 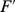 close to zero, and neither potentiation nor depression. Figure 6B shows the results of similar simulations for a range of pre-post intervals, applying 60 spike pairings performed at 1 Hz. The Convallis rule reproduces a STDP curve similar to bi-exponential form found in many computational models [51].

STDP does not fully summarize the nature of cortical synaptic plasticity, which cannot be explained by linear superposition of effects caused by individual spike pairs. Various *in vitro* pairing protocols, in hippocampus [52] or in cortex [26], [53], [54] showed that LTP and LTD pathways can not be reduced to additive interactions of nearby spikes. Therefore, we next asked whether the Convallis rule would also be able to predict additional experimental results beyond STDP. As one of the pieces of evidence in favor of the original BCM theory is the dependence of the sign of plasticity on the rate of tetanic stimulation, we asked if the Convallis rule could produce a similar result. To simulate extracellular stimulation *in vitro*, we synchronously simulated multiple excitatory and inhibitory presynaptic synapses at a range of frequencies ranging from 0.1 Hz to 100 Hz, and investigated the amount of plasticity produced in a downstream neuron. Consistent with experimental data in cortical [55] as well as hippocampal [56] slices *in vitro*, low frequencies resulted in depression while higher frequencies resulted in potentiation (Figure 6C). As a second example, we considered spike triplets in paired recordings (see Materials and Methods). Linear superposition of STDP would predict that presentation of post-pre-post spike triplets should cause no synaptic change; experimentally however, this causes robust potentiation (although pre-post-pre triplets do not) [52]. The Convallis rule is able to reproduce this finding (Figure 6D). A third example of nonlinear plasticity effects concerns the spike pairing repetition frequency. In cortical slices, post-pre pairings at low repetition rates cause synaptic depression, but this converts to potentiation for fast enough repetition rates, a non-linear effect that likely reflects subthreshold phenomena [26]. The Convallis rule produces a similar effect (Figure 6E, top). For pre-post pairings, potentiation is not seen experimentally at low (0.1 Hz) repetition rates in L5 of juvenile cortex [26]. The Convallis rule also replicated this finding (Figure 6E, bottom); for this, the shrinkage mechanism was critical (data not shown). Finally, we asked whether network-level plasticity using the Convallis rule left traces similar to those seen experimentally *in vivo*. Specifically, we assessed whether simulated neurons with similar receptive fields would exhibit higher connection probabilities, as has been reported in mouse visual cortex [57], [58]. This was indeed the case (Figure 6F), strongly for Convallis (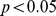, t-test), weakly for rcSTDP (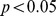, t-test), but not for rate constraint alone. We therefore conclude that the Convallis rule is consistent with a wide range of plasticity phenomena described *in vitro* and *in vivo*, supporting the possibility that a similar process occurs in cortex.

**Figure 7:**
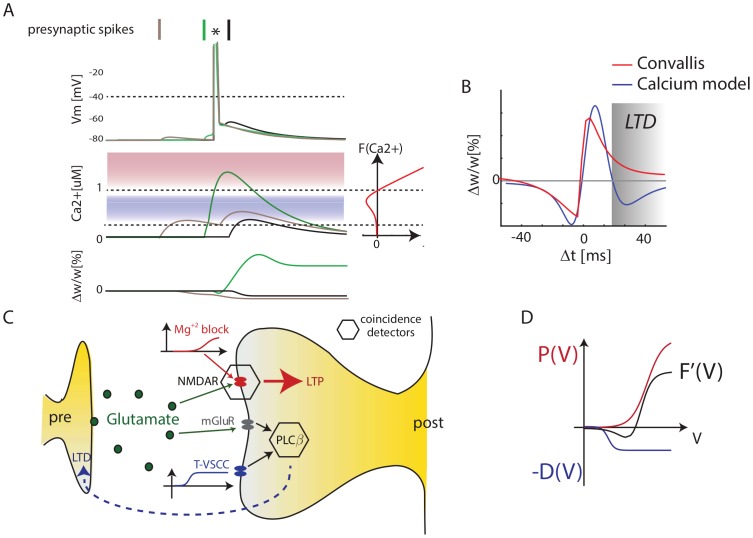
The Convallis rule is inconsistent with the Calcium Hypothesis but consistent with a dual-sensor model. (**A**) Illustration of the calcium hypothesis. In this scheme, the direction of synaptic plasticity depends on calcium concentration, with high concentrations leading to LTP and lower concentrations leading to LTD. The calcium hypothesis predicts that short pre-post pairings produce LTP (green), short post-pre pairings predict LTD (black), but unlike the Convallis also predicts that long pre-post pairings should produce LTD (gray). (**B**) Triphasic STDP curve predicted by the Calcium hypothesis, set against prediction of the Convallis rule. (**C**) Hypothesized cellular mechanism for Convallis rule. LTP is induced by coincidence detection via an NMDA receptor, requiring glutamate and strongly depolarized membrane potential. LTD is induced by a separate coincidence detector with a lower voltage threshold, in which activation of phospholipase C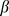 requires coincident activity of group I metabotropic glutamate receptors and T-type (low threshold) calcium channels. (**D**) Summation of the voltage-dependence curves for high-threshold potentiation and low-threshold depression gives the 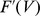 function of the Convallis rule.

If cortical neurons do indeed implement a rule similar to Convallis, what cellular mechanisms might underlie it? Plasticity in the developing neocortex appears to involve different cellular mechanisms to those of the well-studied hippocampal Schaffer collateral synapse. One of the leading mechanistic models of hippocampal synaptic plasticity is the calcium concentration hypothesis [59]–[61]. In this model, both LTP and LTD are triggered by calcium influx through NMDA receptors, with LTP triggered by high Ca^2+^ concentrations, and LTD triggered by low concentrations (see Figure 7A). This model has a similarity with Convallis in that weak activation causes LTD and strong activation LTP. Nevertheless, the functional form of the Convallis rule (Eqn. 1) has a critical difference to the calcium hypothesis. In the Convallis rule, the nonlinear function 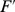 that determines the sign of synaptic plasticity operates directly on the membrane potential prior to coincidence detection with presynaptic input, whereas in the calcium rule this nonlinearity happens after coincidence detection. This leads to a diverging experimental predictions, with the calcium model predicting a triphasic STDP curve [60] (but see also [61]). This has been reported in some hippocampal experiments [62], [63], but not in the neocortex (Figure 7B).

A substantial body of experimental evidence suggests that in juvenile neocortical neurons, the potentiation and depression components of STDP are produced by different cellular mechanisms [27]–[33]. While these data are obtained from different sensory cortices (visual, somatosensory), and for different cortical synapse types (typically L4→L2/3 or L5→L5), they suggest a hypothesis for a common mechanism underlying STDP in at least some neocortical synapses [6]. In these systems, LTP appears of the conventional type, dependent on postsynaptic NMDA activation caused by coincident glutamate release and release of magnesium block by postsynaptic depolarization. For LTD however, induction is independent of postsynaptic NMDA receptors, and instead appears to be induced by a separate mechanism in which postsynaptic phospholipase C*β* acts as a coincidence detector for the activation of group I metabotropic glutamate receptors, and postsynaptic depolarization detected by voltage-sensitive calcium channels (VSCCs), leading to presynaptic expression of LTD via retrograde endocannabinoid signaling. Importantly, the VSCCs implicated are of the low-threshold T-type [27], [30]. Together, these results suggest a hypothesis that in the developing sensory cortex, there exist two separate molecular coincidence detectors for LTP and LTD, and that the coincidence detector for LTD has a lower voltage threshold (Figure 7C; [6], [32].

The mathematical form of the Convallis rule is consistent with just such a mechanism. The function 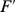 can be expressed as a difference of two non-negative functions 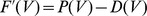, both sigmoidal in shape, but with 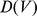 having a lower threshold. The rule can then be expressed as a sum of two terms
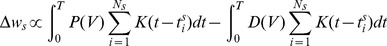
This equation has a natural mechanistic interpretation, as the result of two coincidence detectors. The first, corresponding to 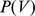, is activated when the membrane is strongly depolarized after a presynaptic spike fires, and leads to synaptic potentiation. The second, corresponding to 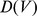, is activated when the membrane is moderately depolarized after presynaptic firing, and leads to synaptic depression. Linear addition of 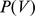 and 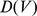 would be expected due to their implementation by separate coincidence detectors, triggered by spatially separated calcium sources [64]. The mathematical form of the Convallis rule therefore bears a striking resemblance to a leading hypothesis for the mechanisms synaptic plasticity in the juvenile sensory cortex.

## Discussion

We derived a synaptic plasticity rule for unsupervised learning in spiking neurons, based on an optimization principle that increases the skewness of subthreshold membrane potential distributions, under the constraint of a fixed mean firing rate. Applying this rule to a speech recognition task caused individual neurons to develop skewed membrane potential distributions and selective receptive fields both in a feedforward configuration and within a recurrent network. The spike count outputs of the recurrent network were sufficient to allow good readout by a linear classifier, suggesting that this unsupervised rule had enabled the network to form an easily-decoded representation of the key spatiotemporal features of the input that distinguished the spoken digits. Simulation of paradigms used to study synaptic plasticity *in vitro* produced similar behaviour to that found experimentally. Furthermore the form of the rule is consistent with a dual-sensor mechanism that has been suggested experimentally for cortical neurons.

The phenomenon of spike-timing dependent plasticity has been robustly observed in a large number of neuronal systems (see for example [65] for review). It is important to remember however that STDP is not a fundamental description of synaptic plasticity, but simply an experimental observation that describes how synapses respond to one particular stimulus of temporally offset spike pairings [3]–[6]. We found that the Convallis rule, when presented with paired spikes, reproduced a biphasic STDP curve. However, implementation of all-to-all STDP alone produced both a worse fit to experimental plasticity paradigms, and poorer unsupervised learning of speech sounds than the full Convallis rule. Implementation of other learning rules described in the literature which match more experimental observations than STDP alone [21], [23] also produced poorer results.The higher performance of Convallis compared to rules based on spike timing alone may reflect the fact that the subthreshold potential conveys additional information that is useful to guide synaptic plasticity. We note however that better unsupervised learning was also obtained compared to a previous phenomenological rule [23] that exhibited a similar voltage dependence, but was derived primarily to match experimental observations, rather than derived from an optimality principle. Other than the similar voltage dependence, this rule was different in many details to Convallis, for example with regard to the precise temporal relationship of presynaptic activity and postsynaptic voltage required for potentiation or depression. The derivation of these relationships from an optimality principle might underlie Convallis' better performance. Additionally or alternatively, the difference might reflect a difference in the stabilizing mechanism between the two rules. For Convallis, we found that a penalty-based weight decay term could not provide optimal stability, and much better performance was obtained with a hard constraint on firing rate with plasticity inhibited until the constraint was satisfied. In our simulations of the framework of [23], we were similarly unable to obtain robust stabilization of firing rates, which may have contributed to poorer learning performance.

Although unsupervised learning has long been proposed as a primary function of the sensory cortex [12], [13], the circuit mechanisms underlying it are still unknown. One influential class of models holds that unsupervised learning occurs through the coordinated plasticity of top-down and bottom-up projections, leading to the development of “generative models” by which the brain learns to form compressed representations of sensory stimuli [66]–[68]. Although these models have produced good performance in real-world tasks such as optical character recognition, the mapping between these abstract models and concrete experimental results on cortical circuitry and plasticity is as yet unclear, and their implementation in spiking neuron models has yet to be demonstrated. Here we describe an alternative scheme for unsupervised learning in cortex, in which every neuron acts essentially independently, using a plasticity rule to form an unsupervised representation of its own synaptic inputs. Despite the simplicity of this approach, it could be applied in recurrent spiking networks to produce good unsupervised learning. We hypothesize that incorporating other mechanisms to coordinate plasticity at the network level [69] may further improve network performance.

In psychophysical experiments, perceptual learning is typically studied by repeated practice at sensory discrimination tasks. In such cases, learning might be boosted by attention directed to the stimuli to be learned, or rewards delivered after a correct response. Nevertheless, purely unsupervised perceptual learning can also occur in humans, both in development [8] and adulthood [70]. The Convallis rule as simulated here is a purely unsupervised rule that operates continuously. The effects of attention, reward and task-relevance could be captured in the same framework by a modulation of learning rates by neuromodulatory tone [71], [72]. This would allow cortical networks to devote their limited resources to representing those stimulus features most likely to require behavioural associations.

Models of synaptic plasticity typically fall into three classes: phenomenological models, which aim to quantitatively summarize the ever-growing body of experimental data [21]–[23]; mechanistic models, which aim to explain how these phenomena are produced by underlying biophysical processes [60], [73]; and normative models, which aim to explain the information-processing benefit that synaptic plasticity achieves within the brain [74]–[79]. The Convallis rule bridges all three levels of analysis. Being mathematically derived from an optimization principle, it belongs in the normative class, and the fact that it can organize recurrent spiking networks to perform unsupervised learning in a real-world task supports the idea that a similar principle could enhance cortical information processing. The rule is consistent with a number of experimental findings on cortical plasticity, including but not limited to STDP, suggesting that a similar principle may indeed operate in cortical cells. Finally, the functional form of the Convallis rule has a direct mechanistic interpretation in terms of a dual coincidence-detector model, for which substantial evidence exists in neocortical synapses [27]–[32], [32,33]. Based on this confluence of normative, phenomenological, and mechanistic evidence, we suggest that the Convallis rule may approximate a fundamental computational principle of the neocortex.

## Materials and Methods

### Neuron model

Simulations of the spiking neurons were performed using a custom version of the **NEST** simulator [80] and the **PyNN** interface [81], with a fixed time step of 0.1 ms. In all simulations, we used an integrate-and-fire neuron model with a membrane time constant 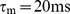, a leak conductance of 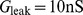, and a resting membrane potential 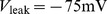. Spikes were generated when the membrane potential 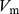 reaches the threshold 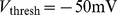. To model the shape of the action potential, the voltage was set to 20 mV after threshold crossing, and then decayed linearly during a refractory period of time 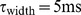 to a reset value of 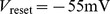, following which an exponentially decaying after-depolarizing current 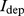 of initial magnitude 50 pA and time constant 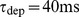 was applied. We used this scheme with a high reset voltage and ADP, rather than the more common low reset value, as it provided a better match to intracellular recordings *in vitro* and *in vivo* (see supplementary Figure S1). Synaptic connections were modelled as transient conductance changes with instantaneous rise followed by exponential decay. Synaptic time constants were chosen to be 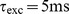 and 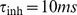 for excitation and inhibition respectively, and reversal potentials were 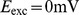 and 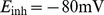.

The complete set of equations describing the dynamics of a neuron is thus given by
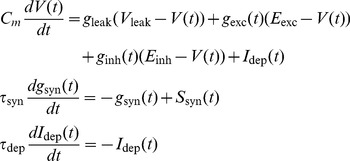
(2)where 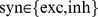, 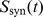 are the incoming synaptic spike trains represented as sums of delta functions.

### Learning rule

In the Convallis rule, a neuron adapts its synapses in order to optimize an objective function 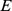 depending on its membrane potential 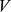:
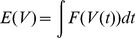
(3)


To enforce skewness of the distribution of postsynaptic potentials, we chose an objective function that penalized intermediate membrane potential values, but rewarded membrane potentials close to either resting potential or spike threshold. Because the neuron spent considerably less time depolarized than hyperpolarized, the objective function was chosen to reward potentials close to spike threshold more strongly than potentials close to rest. For all simulations in the present paper, we used a sum of a logistic function and of its integral. More precisely:
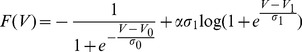
(4)Parameters values were taken as *V*_0_ = −55 mV, *V*_1_ = −52 mV, *σ*_0_ = 4 mV, *σ*_1_ = 2 mv and 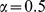, and the same parameters were used for both the speech processing application and simulation of *in vitro* experiments. The shape of 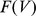 was therefore constant in all the simulations of the paper, and its exact form did not appear to be crucial (as long as a clear valley-shaped function was used), since similar results were achieved with a variety of functions (not shown).

To derive the Convallis rule, we used a gradient ascent method. Differentiating 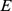 with respect to incoming synaptic weights 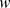 gives
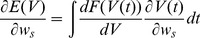
(5)


To compute 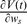, we considered the variable 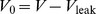. Equation 2 can be rewritten as
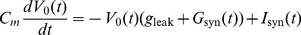
(6)


Where 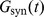 is the total synaptic conductance and 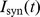 the synaptic current. Specifically, if 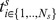 are the times at which a particular synapse 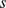 of weight 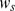 is active, and if 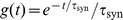 (if 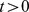) is the kernel function representing the conductance time course,
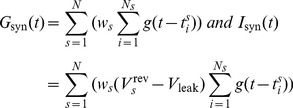
(7)where 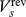 is the reversal potential of synapse 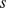. Inspecting equation 6, we see that for a conductance-based neuron, 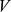 integrates 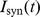 with an effective time constant 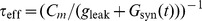. Approximating 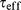 by a constant equal to 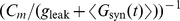 where 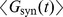 denotes a running average of the synaptic conductance [82], we can approximate 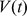 by the following equation:
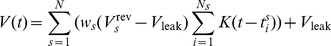
(8)where
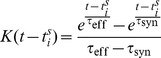
(9)Note that this approximation holds as long as we ignore the reset mechanism and non-linearity due to the spike, an approximation that will be more accurate when using a “soft” reset mechanism as described here. Substituting in equation 5, we obtain the following equation for the gradient:
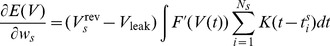
(10)


This generic form is similar to previous supervised learning rules that were also based onto the post-synaptic 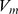, such as the Tempotron [82], [83] or Chronotron [84]. As noted by [85], 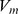 is used here as a proxy for the input current flowing into the cells, which is the only relevant quantity at the cell level to measure the correlation between incoming pre and post-synaptic activity.

To prevent plastic changes for spurious single pairings, plasticity changes are accumulated through the convolution of a slowly decaying exponential, and then expressed at the synapse level only if the accumulated value crosses thresholds 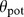 and 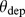 for respectively potentiation and depression. Specifically, we define
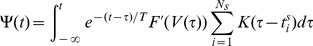
(11)


The time constant 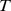 of the slowly decaying exponential is taken to be 1 second throughout the paper. The final weight changes are then given by
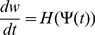
(12)where the shrinkage function 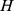 is defined as
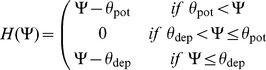
(13)


Throughout the paper, we fixed the values of 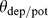 to −10 and 50 respectively. A graph of 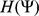 can be seen in Figure 1C. Note that the weights are clipped to hard bounds values 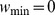
nS and 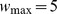
nS. The Convallis rule has therefore have 3 parameters in addition of the shape of 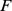: the time at which the changes are accumulated 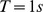, and those two thresholds for the shrinkage function.

### Implementation

Direct calculation of the above integrals would be prohibitive in large-scale simulations, as it would require computing the products 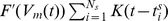, for all synapses and for each time step, resulting in a complexity scaling in 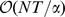, where 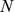 is the number of synapses, 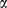 the time step, and 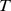 the simulation length. To speed up implementation of the algorithm, we write:
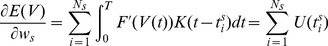
(14)where 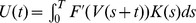. We can implement the rule much faster by first computing and storing the history 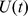 for neuron, and computing weight changes as a sum over all input spikes 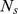 for all synapse 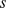, which is of order 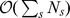. To compute 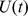, we note that 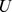 is the convolution of 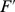 and a filter 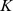 which is a difference of decaying exponentials (see Equation 9). By defining 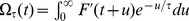, we can write 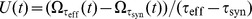. Integrating by parts, we obtain
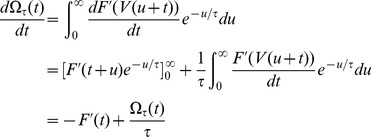
Therefore, we have a differential equation that can be used to compute look-up tables of 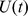 for all neurons during this period, by running backwards in time from starting values 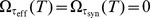. Weight changes are then calculated by summing over spikes. We note that this method of running backward in time is simply a trick to speed up execution time, and is equivalent to the original deterministic algorithm. In practice, we perform this by stopping the simulation after the presentation of each input pattern (T = 1 s). This implementation does not impact the results when the frequency 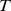 of the updates is changed (data not shown), as long as the assumption 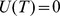 is valid, which will hold provided the support of the 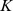 filter is shorter than 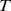.

### Firing rate constraint

Run in isolation, the above rule is unstable, as the response of the neuron tends to accumulate either above or below the plasticity threshold, leading to either explosive increases in synaptic weights or convergence of all weights to zero. In the BCM theory, this problem was solved by a sliding plasticity threshold, computed as a long-running average of the firing history of the post-synaptic neuron. For the Convallis rule we found that a sliding threshold was not necessary, provided a mechanism was in place to constrain the neurons firing rate to a fixed value. We implemented this via “synaptic scaling” [86], using an approach analogous to the projected subgradient method for constrained optimization. In the projected subgradient method, gradient-following steps are allowed to temporarily break the constraint, but are followed by a projection onto the constraint subspace. Because direct projection onto the subspace of synaptic weights corresponding to the targeted mean firing rate would not be computationally tractable or biologically realistic, we instead used a Proportional-Integral (PI) controller [87] to enforce the constraint, and suppress gradient learning until the constraint was re-established. Specifically, we define 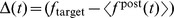 to be the deviation from target mean firing rate, where 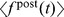 is a cell's firing rate computed as a running average over its past-history with a time constant T (10 s in our simulations) and 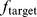 is the targeted mean rate. The output of the PI controller is
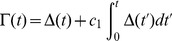
where 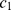 is a coefficient regulating the contribution of the integral term. The value of 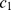 balances speed of convergence against the possibility of oscillation; in all simulations, we fixed 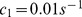. To suppress gradient descent until the constraint was satisfied, we scaled the synaptic plasticity rule by a term 
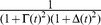
 that was small if either 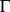 or 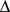 was not close to zero, leading to a final form of
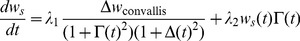
(15)


The parameters 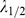 were set to 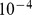 and 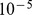, respectively. We found this latter feature was essential for stable operation of the Convallis rule.

### Circular Gaussian simulations

In simulations of artificial data (Figure 2), 1000 excitatory and 250 inhibitory inputs were connected to a single post-synaptic neuron. Only excitatory connections were plastic. Initial values of the weights were drawn from Gaussian distribution 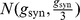 with 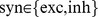. The values were 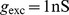 and 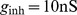, and the target output rate was fixed to 10 Hz. Pre-synaptic neurons were stimulated with wrapped Gaussian profiles of rates 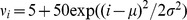 spikes/sec, the centre 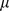 being shifted randomly every 100 ms over all possible positions 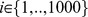 and with 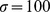. The tuning index used in Figure 2 was computed as a directional statistic: for each cell, the distance between neuron 0 and 1000 was mapped into an angle 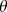, and if 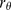 is the average firing rate for this particular angle, the tuning was defined as 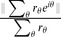. The closer the tuning is to 1, the more the neuron is responding only to one particular angle.

### TIDIGITS database

To test the ability of the rule to perform unsupervised learning in a real-world context, we applied it to a problem of speech recognition, using the TIDIGITS database [40]. This data consists of recordings of eleven English digits (“zero” to “nine” plus “oh”), spoken twice each by 326 speakers of various ages and genders (man, woman, boy, girl), at a sampling rate of 20 KHz. The TIDIGITS database was separated into its standard training and test sets of 167 speakers each. The raw recorded waveforms were pre-processed into spike trains using the Lyon model [41], to produce a simulated cochleogram of 93 frequency channels. The cochleogram output for each digit was centered in a one second epoch, sampled at 500 Hz, and normalized to equalize the summed activity of all frequencies for all digit utterances. Input spike trains were generated as inhomogeneous Poisson spike trains with intensity function given by the cochleogram output, at an average frequency of 5 Hz.

For feedforward simulations (Figure 3), each target neuron received plastic excitatory projections from 50% of randomly chosen cochleogram cells with initial conductances 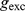 uniformly drawn in [0, 10 nS] and synaptic delays uniformly drawn from [0.1 ms,5 ms], while also receiving static inhibitory projections from all cells in the cochleogram with conductances 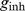 uniformly drawn in [0, 40 nS].

For recurrent network simulations, 4500 neurons were simulated with an excitatory/inhibitory neuron ratio of 4∶1 on a square sheet with periodic boundary conditions. Every neuron was sparsely connected with the rest of the network with a connection probability of 5%. Synaptic delays were drawn randomly from a uniform distribution between 0.1 and 5 ms. Initial synaptic conductances were taken randomly from Gaussian distributions with means 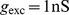 and 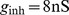, and standard deviations equal to a third of their means. To sustain spontaneous activity, each neuron also received an independent Poisson spike train at a frequency of 300 Hz, through an excitatory synapse of weight 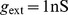. Although recurrent connections were uniform, input connections were arranged in a tonotopic manner, with each cochleogram cell projecting with excitatory synapses to a fraction of 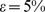 of neurons in the network, with a probability following a Gaussian profile 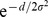 (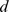 being the distance between the source and a target neuron within the network, and 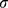 being equal to 0.2 unit). The mean conductances of the external connections were equal to the recurrent ones, i.e 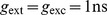, and all external inputs were fixed rather than plastic.

To measure the selectivity of a neuron to the digit stimuli, we used the F-statistic, commonly used in one-way analysis of variance (ANOVA). Specifically, to measure the difference between mean spike counts of each digit, relative to within-digit variance, we computed
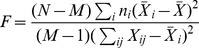
(16)where 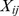 is the spike count the neuron produces on the 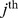 presentation of digit 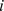, 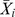 is the mean response to digit 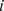, 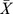 the overall mean response, 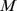 the number of digits, and 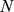 the total number of stimulus presentations.

To quantify the efficacy of unsupervised learning, we evaluated the ability of a downstream linear classifier to identify the digit spoken from the spike counts of each simulated neuron. This approach therefore evaluates the network's ability to form a linearly separable representation of the digit inputs that can be read out without requiring temporal analysis. Specifically, if 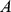 is a matrix of size 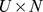 containing the mean firing rate of all 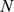 cells to each of the 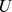 digit utterances in the training set, and if 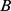 is an “answer” matrix of size 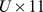 with each row consisting of all zeros except a single 1 indicating the presented digit during this trial, we used multi-class linear support vector machine [88] to find a matrix 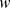 of size 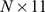 to predict B from A. Performance was evaluated by computing 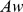 on the test set, and classifying each utterance according to the highest value. The cost parameter 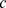 used for the support vector machine was set to 0.01. We note that while the SVM was for efficiency trained with a (biologically unrealistic) quadratic programming algorithm, the same solution would be found by the perceptron rule [42]. Ridge regression learning was also tried (data not shown), leading to qualitatively similar results.

### Comparison with other learning rules

Throughout the paper, the rcSTDP rule is implemented as a normal additive STDP rule combined with the PI mechanism described for the Convallis rule (Equation 15), in order to ensure that the same output firing rate is achieved. Optimization of this rule's parameters is described in Figure S2. To compare the Convallis rule with NN-STDP (STDP with interactions only between neighbouring pairs of spikes [20]) or triplet STDP [21], we again combined these rules with a PI mechanism to make sure that they were stable and had the same rate constraint. For the rule of [23], we did not add the firing rate constraint, as it already contains a homeostatic mechanism. In all cases, we used the parameter values in the originally published manuscripts; in the case of the triplet rule, we used the data obtained from the fit to visual cortex data.

### Simulations of *in vitro* experiments

For all *in vitro* simulations (except Figure 6C), we considered only two neurons with a single connection between them. The parameters used for the learning rules were the same as in the learning applications. The initial synaptic strength of the connection, if not specified elsewhere or varied, was taken to be 2 nS. All parameters had the same values as in the network simulations, but since it is assumed that these *in vitro* protocols are taking place over a short time scale, the rate constraint mechanism of the model was turned off. For Figure 6C, we considered a group of 20 excitatory and 5 inhibitory synapses, connected onto a single post-synaptic neuron. For each stimulation of the simulated afferent fibers, every synapse had 50% chance of being active. The fibers were stimulated with 100 presynaptic pulses at varying frequencies, as in *in vitro* experiments [55]. To reproduce the triplet experiment [21], [52], we use a stimulation protocol of 60 triplet of spikes repeated at 1 Hz. Each triplet consists of two pre and one post synaptic spikes or two post and one pre-synaptic spikes, as can be seen in the inset of Figure 6D (see references for more details). To reproduce the dependance on frequency [26], we used a protocol as in the original paper: interdigitated burst of 5 spikes paired with a given 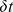 and frequency repeated 15 times at a 0.1 Hz frequency, thus leading to 75 spikes in total.

## Supporting Information

Figure S1 **Reset mechanism.** In integrate-and-fire neuronal simulations, the membrane potential is often reset to its resting value after each spike. Although this might be an appropriate model of certain neuronal classes, cortical pyramidal cells do not show this behavior. Instead, pyramidal cells return to a voltage only just below spike threshold after an action potential is fired, and frequently exhibit an after-depolarization caused by activation of dendritic voltage-gated conductances, which is believed to underlie burst firing [34]. (**A**) Intracellular recording trace of a L5 pyramidal cell in mouse visual cortex (courtesy of M. Okun). Note the lack of reset to resting potential after spike firing. (**B**) Illustration of membrane potential trace generated in response to white noise injection by a neuron with hard reset to resting potential after spike firing. Note the clear difference in reset behavior to the data in (A), and the lack of burst firing. (**C**) Illustration of membrane potential trace generated in response to the same input, by a neuron with soft reset to −55 mV after spike firing and ADP (the model used in all simulations). Note the more realistic spike reset and presence of burst firing.
(EPS)

Figure S2
**Calibration of the rcSTDP rule.** (**A**) To obtain optimal performance with the rcSTDP rule, we performed a parameter search varying the the rate constraint parameter 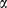 and the STDP learning rate 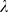, initially on a linear scale, for the wrapped Gaussian stimulus ensemble. Performance was assessed as the skewness of the final 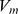 distributions, shown in the pseudocolor matrix presented. Note the peak for values around 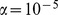 and 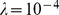. (**B**) To gain further accuracy we performed an additional parameter search fixing 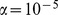, with 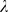 now on a log scale. A peak was seen at 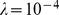.(EPS)

Figure S3 **Additional details of Convallis performance, feedforward case.** (**A**) Weight distribution after learning the speech data. Note that Convallis leads to a highly skewed distribution with a large mode at 0 and a secondary peak at larger values, corresponding to a sparse weight matrix consisting of mainly silent synapses. STDP by contrast leads to a single-peaked distribution. (**B**) Convergence analysis. To show that all rules had converged we plotted the mean-square weight change in weight between consecutive training iterations. For all rules, the mean change tended to zero, indicating that weights had converged. (**C**) To evaluate whether the Convallis rule had detected true temporal features, rather than simply power in different frequencies, we evaluated performance on time-reversed digit stimuli. The unsupervised representation was trained using forward presentations only, and spike counts were measured in response to time-reversed digits. These spike counts were then fed into the SVM classifier to predict the presented digit. Classification performance was poorer, even when the SVM was retrained on the spike counts generated in response to time-reversed digits. This indicates that the Convallis rule has produced an unsupervised representation of temporal features in the input stimulus, rather than just frequency selectivity.
(EPS)

Figure S4 **Comparison to alternative learning rules, feedforward case.** In addition to rcSTDP, whose performance is shown in the main text, we also compared the Convallis rule to various other learning rules described in the literature, specifically nearest-neighbor STDP (NN-STDP) [20], triplet STDP [21], and a rule based on post-synaptic voltage [23]. This figure shows the same analyses as Figure 4 for these rules. (**A**) Histogram of subthreshold potentials for the cell illustrated in Figure 3, accumulated over all test-set data after learning with the three alternative plasticity rules. (**B**) Distribution of skewness for 4500 neurons trained similarly from random initial weights. Note that skewness after Convallis training is markedly higher than after the rcTriplet, NN-rcSTDP, or Clopath rules. (**C**) Mean rate response of the same example neuron to all digits. Errors bar show s.e.m. (**D**) The strength of tuning for each neuron was summarized by an F-statistic that measured the selectivity of its spike counts for particular digits (see Materials and Methods). The main graph shows a histogram of tuning strength across the simulated population for the 3 learning rules and the raw cochleogram input, while the inset shows mean and standard error. Again, Convallis shows greater selectivity. (**E**) To evaluate the ability of these rules to perform unsupervised learning, the spike count responses of up to 4500 cells were used as input to a linear classifier trained to distinguish digits. (**F**) Mean classification performance as a function of the number of unsupervised neurons. (Errors bars show s.e.m over 10 independent runs of the analysis). Note that while the alternative rules exhibit better performance than rate constraint alone, they do not match the Convallis performance.
(EPS)

Figure S5 **additional details of Convallis performance, recurrent case.** (**A**) Weight distribution after learning the speech data. As in the feedforward case, the Convallis rule exhibits a sparse weight distribution while STDP produces a single-peaked distribution. (**B**) Convergence analysis showing the mean-square weight change in weight between consecutive training iterations. For all rules, the mean change tended to zero, indicating that weights had converged. (**C**) To evaluate whether the Convallis rule had detected true temporal features, rather than simply power in different frequencies, we evaluated performance on time-reversed digit stimuli. The unsupervised representation was trained using forward presentations only, and spike counts were measured in response to time-reversed digits. These spike counts were then fed into the SVM classifier to predict the presented digit. Classification performance was poorer, even when the SVM was retrained on the spike counts generated in response to time-reversed digits. This indicates that the Convallis rule has produced an unsupervised representation of temporal features in the input stimulus.
(EPS)

Figure S6 **Comparison to alternative learning rules, recurrent case.** To evaluate how other plasticity rules from the literature operated in a recurrent framework, we attempted to simulate the same plasticity rules as in Figure S4. Although the triplet and NN-STDP rules (supplemented with the firing rate constraint) were stable and fast enough to simulate in our recurrent network of 4500 cells, we were not able to simulate the rule of [23] as we found large networks implementing this rule were unstable. (**A–C**) Distributions of ISI CVs, firing rates, and pairwise correlation coefficients (averaged over 2000 randomly chosen pairs of cells) in the network before and after learning with rcTriplet and NN-rcSTDP rules. Note that none of the learning rules produce a change in any of these measures of network dynamics. Error bars show the standard deviation. (**D**) Distribution of membrane potential skewness for 200 randomly chosen cells in the network before or after learning. Note that skewness is highest with the Convallis rule. (**E**) Distribution of the tuning sharpness (as measured by F-statistic) for all neurons before and after learning. Inset displays the mean of the distributions. Error bars show standard deviation. (**F**) Classification performance as a function of the number of neurons considered by the external classifier, for various learning rules. Errors bars show s.e.m over 10 different simulations, run independently from different random seeds. Neither rule gave significantly improved performance over rate constraint alone (p>0.05).
(EPS)
